# Factors Affecting Root Canal Treatment Case Difficulty, Practitioner Rating of Difficulty and Treatment Complications Among General Dentists and Endodontists: A Prospective Cohort Study From National Dental Practice‐Based Research Network PREDICT Project

**DOI:** 10.1111/iej.70019

**Published:** 2025-08-22

**Authors:** Alan S. Law, Sanket Nagarkar, Ellen Funkhouser, Rahma Mungia, Donald R. Nixdorf, Ernest W. N. Lam, Ali Nosrat, Robert S. Roda, Gregg H. Gilbert, National Dental PBRN Collaborative Group (The National Dental PBRN Collaborative Group comprises practitioners, faculty and staff investigators who contributed to this network activity), Tracy Shea, Stephanie Hodge, Christine O’Brien, Hanna Knopf, Deborah McEdward, Shermetria Massengale, Stephanie Reyes, Meredith Buchberg, Colleen Dolan, Andrea Mathews, Terri Jones

**Affiliations:** ^1^ Specialty Practices Roseville Minnesota USA; ^2^ Division of Endodontics University of Minnesota Minneapolis Minnesota USA; ^3^ Park Dental Partners Coon Rapids Minnesota USA; ^4^ Department of Prosthodontics and Crown and Bridge Dr. D. Y. Patil Dental College, Dr. D. Y. Patil Vidyapeeth Pune Maharashtra India; ^5^ Department of Restorative Sciences, School of Dentistry University of Minnesota Minneapolis Minnesota USA; ^6^ Division of Preventive Medicine, School of Medicine University of Alabama Birmingham Alabama USA; ^7^ Department of Periodontics, School of Dentistry The University of Texas Health San Antonio Texas USA; ^8^ Division of TMD and Orofacial Pain University of Minnesota Minneapolis Minnesota USA; ^9^ University of Toronto Faculty of Dentistry Toronto Ontario Canada; ^10^ Division of Endodontics, Department of Advanced Oral Sciences and Therapeutics, School of Dentistry University of Maryland Baltimore Maryland USA; ^11^ Private Practice, Centreville Endodontics Centreville Virginia USA; ^12^ Private Practice Scottsdale Arizona USA; ^13^ Department of Clinical & Community Sciences, School of Dentistry University of Alabama Birmingham Alabama USA

**Keywords:** case difficulty assessment, complications, endodontists, general dentists, outcomes, root canal treatment

## Abstract

**Aim:**

Successful root canal treatment (RCT) is necessary for managing pulpal and periapical disease. The technical quality of RCT affects its outcome. Recognising complicating factors can be important to optimising outcomes. The aims of this study were to compare the practitioner reported case difficulty before and after completion of RCT; and to determine whether any case difficulty items were associated with complications encountered while performing the RCT.

**Methodology:**

One hundred and four general dentists (GDs) and 49 endodontists enrolled 1860 patients needing RCT, April to September 2017. Both before RCT and upon its completion, practitioners used a 10‐point scale to rate the RCT's case difficulty (Difficulty Rating [DR]). Then they used a modified Case Difficulty Assessment Form (CDAF) to record the items for difficulty using the form's list of 10 provided choices. We related practitioners' pre‐RCT CDAF items to their DR ratings and to the procedural complications that they subsequently experienced during this RCT. General estimating equations (GEE) were used to assess the significance of differences in proportions between GDs and endodontists. Non‐parametric tests were used to analyse compositive variables.

**Results:**

Data of 1,698 patients were available for CDAF analyses. The mean CDAF was higher for endodontists than GDs (*p* < 0.001). Pre‐ and post‐RCT DRs were significantly correlated (*r* = 0.79, *p* < 0.001), as were pre‐RCT DR and the number of reported CDAF difficulties (*r* = 0.57, *p* < 0.001). Overall, practitioners encountered complications in treating 16% of patients. The complications were length of obturation > 2 mm from radiographic apex or beyond apex, canals not negotiable within 2 mm of apex, instrument separation, inadvertent filing/file placement past root apex, and perforation. Several CDAF items were independently predictive of complications. Despite the higher CDAF in teeth treated by endodontists, complications were less frequent among endodontists compared to GDs (13% vs. 19%, *p* < 0.001).

**Conclusions:**

Pre‐RCT assessments predicted intra‐operative difficulties and outcomes. Our work underscores the need for targeted assessment tools and specialised training to improve RCT, especially in complex cases treated by GDs.

## 1 Introduction

The goal of root canal treatment (RCT) is to eradicate infection and pain, promote healing, and preserve the natural tooth and its function (Estrela et al. 2014; Azarpazhooh et al. 2022). Successful RCT involves thorough cleaning and shaping of the root canal system, followed by complete obturation to prevent reinfection (Gulabivala and Ng 2023; Pirani and Camilleri 2023). The technical quality of RCT is a determinant of its outcome, and this has been demonstrated in numerous studies (Hommez et al. 2002; De Chevigny et al. 2008; Marquis et al. 2006; Farzaneh et al. 2004; Friedman et al. 2003). Inadequate execution can lead to persistent infection, inflammation and tooth loss (Ng et al. 2011b, 2011a).

Certain identifiable pre‐operative factors, including anatomical variations such as curved or narrow canals, presence of calcifications in the pulp chamber, resorptions, or previously failed treatments, can compromise RCT quality, leading to suboptimal outcomes (Fezai and Al‐Salehi 2019; Ng et al. 2011b, 2008). Therefore, it is imperative to conduct a thorough pre‐treatment assessment to identify potential difficulties and then be prepared to manage them (Almohaimede et al. 2022; Alamoudi et al. 2020).

There is wide variability in the abilities of practitioners to accurately evaluate the difficulty of RCT cases (Mccaul et al. 2001). Several tools have been formulated to aid in this assessment (Essam et al. 2021; Huang et al. 2024; Ree et al. 2003). In the United States, the most commonly used tool is the Endodontic Case Difficulty Assessment Form (CDAF), developed by the American Association of Endodontists (AAE) (AAE 2024). The CDAF identifies a comprehensive list of diagnostic and treatment parameters that may assist the general dentist (GD) in directing endodontic care (AAE 2024).

A previous PREDICT study showed several major differences in clinical approaches (materials and techniques) between GDs and endodontists when performing RCT (Nosrat et al. 2025). There are no previous prospective practice‐based studies comparing the case difficulty and rates of procedural complications between GDs and endodontists. The objectives of this study were to evaluate tooth and patient characteristics that are frequently cited as being difficult in RCT, to compare the CDAF and case difficulty before and after completion of RCT, and to ascertain whether any CDAF items were associated with complications encountered while performing the RCT. Addressing these objectives should contribute to the body of knowledge on the assessment and management of complex RCT cases and possibly enhance the predictive accuracy of difficulty ratings, ultimately leading to improved endodontic treatment planning, execution, and outcomes.

## 2 Materials and Methods

**FIGURE 1:**
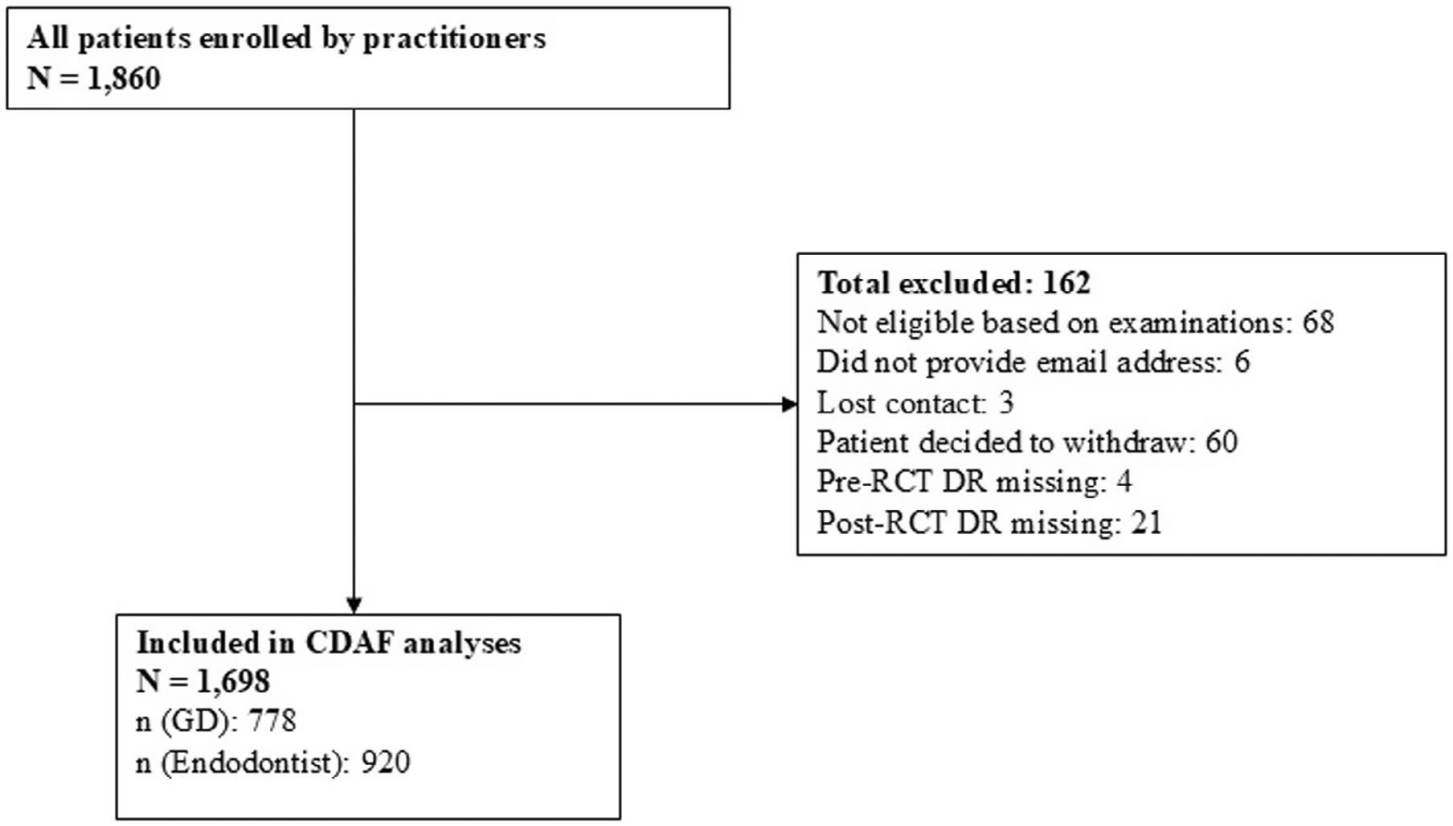
STROBE flow diagram of included patients based on the type of treating practitioner.

This practice‐based prospective cohort study was conducted in the National Dental Practice‐Based Research Network (hereafter referred to as the Network) (Gilbert et al. 2022; Cunha‐Cruz et al. 2023). The University of Alabama at Birmingham (Protocol X161121003) and the University of Toronto (Protocol 34 105) granted human research ethics approval. This observational study follows the principles and guidelines for STrengthening the Reporting of OBservational studies in Epidemiology (STROBE) (Von Elm et al. 2014) (Figure 1) and Preferred Reporting items for OBservational studies in Endodontics (PROBE) (Nagendrababu, Fouad, et al. 2023, 2023).

Patient clinical data were collected by 153 participating practitioners (104 GDs and 49 endodontists) using a consecutive enrollment strategy to avoid selection bias over a 6‐month study enrollment period (April to September 2017). Each GD was asked to enrol up to 7 patients and each endodontist up to 15 patients over a 10‐to‐14‐week period. Study data were collected electronically from patients and practitioners before and after RCT. The full study protocol, practitioner recruitment flyer, and all data forms used in this study are publicly available at: https://www.nationaldentalpbrn.org/recruiting‐ongoing‐upcoming‐completed/#1589296360026‐d0498e9a‐466e. As part of Network membership, practitioners completed an enrollment questionnaire, which elicited information on demographics, practice type/setting, specialty training, and procedures occasionally or routinely performed, including RCT. This questionnaire is publicly available at: https://www.nationaldentalpbrn.org/wp‐content/uploads/2020/05/Network‐EnrollmentQuestionnaire‐2013‐07‐15‐V9‐0‐2.pdf

Patients' eligibility criteria included: 18 or 19 years of age or older (depending on the state); their stated ability to return to the clinic in 12 months; be willing to provide contact information for another person; willing and able to complete questionnaires electronically; provide informed consent; be able to read in English or Spanish; and have a permanent tooth in need of a RCT. Practitioners determined the final eligibility requirements that the tooth had no prior RCT; only one tooth required RCT; and that the RCT was completed.

At both pre‐ and post‐RCT, practitioners recorded a difficulty rating (DR) (0 ‘minimal’ to 10 ‘high’) and the CDAF: ‘Patient has limited ability to open mouth’; ‘The patient's gag reflex adversely impacted obtaining a periapical radiograph’; ‘A crown restoration is present on the treated tooth’; ‘Calcifications are present within the pulp chamber’; ‘The longest root is > 21 mm from the reference cusp tip to the apex’; ‘The curvature of the most curved root is substantial (≥ 20°) or S‐shaped’; ‘Each root has ≥ 1 canal that is not clearly visible for the entire length of the root’; ‘Incomplete root development’; ‘Root resorption is evident (internal, external, or apical)’; and ‘Other (please specify)’. As this was a study of how GD/endodontists use these instruments, there were no training or calibration sessions for CDAF/DR. The numerical DR scale is often preferred over descriptive verbal categories in clinical research because it provides greater granularity and sensitivity, allowing practitioners to more precisely quantify perceptions of case difficulty and detect subtle variations across cases; this enhances statistical power and reliability in data analysis (Streiner and Norman 2008). When the tooth was a molar, this was noted by the practitioner recorded on the pre‐RCT CDAF. Practitioners were only asked to complete the CDAF again after treatment if they had rated the post‐RCT DR at 3 or higher. This threshold was chosen to focus on cases where practitioners perceived meaningful difficulty post‐treatment. Items categorised as ‘Other’ were reviewed by ASL, and where appropriate, were included with an existing item.

The post‐RCT form also included a section on complications that occurred during treatment: ‘Perforation (from chamber or canal) into bone or gingival tissue’; ‘≥ 1 (all) canals not negotiable within 2 mm of radiographic apex’; ‘Inability to complete treatment due to discomfort during treatment’; ‘Separation of instrument in canal space’; ‘Inadvertent filing or inadvertent file placement past the root apex’. Inability to complete treatment was not used as a complication because it only indicated that the RCT was not completed that day. In addition, there was a question on length of obturation with 3 response options: ‘A canal > 2 mm from radiographic apex’, ‘All canals obturated within 2 mm radiographic apex’ and ‘Obturation beyond radiographic apex’. Categories/options that differed from canals being obturated to within 2 mm from the apex were classified as a complication, that is to say, obturation > 2 mm from radiographic apex and obturation beyond radiographic apex.

## 3 Statistical Analysis

Descriptive statistics, mean and standard deviation (SD), median with inter‐quartile range (IQR) were used to describe each characteristic of interest, when appropriate. DRs were analysed as continuous variables. Proportions were used for the analysis of CDAF items. Significance of differences in proportions between GDs and endodontists was ascertained using a logistic regression model that used generalised estimating equations (GEE) that adjusted for clustering of patients within practice, and whether the tooth was a molar, implemented using PROC GENMOD in SAS with CORR = EXCH option.

Nonparametric tests were used to assess the significance of differences in composite variables of number of difficulty items and DR by practitioner type. Spearman rank correlations were used to assess associations of the number of CDAF difficulty items and the DR. The analyses of CDAF items are primarily pre‐RCT because practitioners unless the post‐RCT DR was 3 or higher. The impact of each CDAF item on DR was assessed by calculating the mean difference of DR for the CDAF item that was cited and when it was not. Although the DRs were not normally distributed, the difference in means rather than medians was used because the former better captured the nuance in impact (differences in medians by definition were whole numbers) of the CDAF item. The Wilcoxon rank sum test, a nonparametric test, was used to assess the significance of differences. The differences were assessed individually; then all were entered into one model to assess independence of associations. The Kruskal–Wallis test was used to assess differences jointly, that is, when all in one model. The association of each pre‐RCT CDAF item with each of five RCT complications was ascertained using GEE described above. These associations were adjusted for whether the dentist was an endodontist as an indicator of skill level. Independence of associations was ascertained by including only the CDAF items that had an association with DR at *p* < 0.1 into one model. All analyses were conducted using the SAS (SAS Institute, Cary, NC, USA) v9.4 statistical package.

## 4 Results

A total of 1860 patients were screened, determined to be eligible, and consented by 153 practitioners. Of these, 137 patients who were determined to be eligible were not enrolled: 68 patients were withdrawn by a practitioner because they were deemed not to be eligible based on clinical examination; 6 patients were not eligible because they had moved or did not provide an email contact; 3 were lost to contact; and 60 patients changed their minds and left without completing baseline questionnaires. Pre‐RCT DR was missing for 4 patients, and the post‐RCT DR was missing for 21 patients. Analyses were conducted on 1698 patients with available pre‐ and post‐RCT DRs (Figure 1).

### 4.1 Practitioner Characteristics

The mean (SD) age of the 153 practitioners was 51.3 (11.7) years (median = 52, IQR: 42 to 62). The majority, 73%, were male, 70% non‐Hispanic white, 74% were private practice owners with over half working in solo practices, and 32% were endodontists. The demographics of the GDs and endodontists were similar. Endodontists enrolled over twice as many patients as GDs (mean [SD]: 19.0 [13.7] vs. 7.6 [5.6], *p* < 0.001) and were less likely to be owners of solo private practices (21% vs. 52%, *p* < 0.001). All endodontists routinely performed RCT on premolars and molars; fewer GDs did (92% for pre‐molars and 71% for molars) (Table 1).

**TABLE 1:** Practitioner characteristics, pre‐RCT case difficulty assessment form (CDAF) reasons, and pre‐ and post‐RCT difficulty ratings (DR), overall and by whether the dentist performing the RCT was a general dentist or an endodontist.

Characteristics	All		General dentist		Endodontist		*p* ^a^
	*N*	%	*N*	%	*N*	%	
Practitioner characteristic	*N* = 153		*N* = 104		*N* = 49		
Male	111	73%	75	72%	36	74%	0.9
Age: mean (SD)	51.3 (11.7)		52.1 (11.8)		49.5 (11.2)		0.2
Non‐Hispanic White^b^	105	70%	71	70%	34	71%	
Practice type							
Solo private practice	63	42%	53	52%	10	21%	< 0.001
Owner private practice	48	32%	22	22%	26	54%	
Other practice settings^c^	39	26%	27	26%	12	25%	
Number of patients enrolled: mean (SD)	11.2 (10.4)		7.6 (5.5)		19.1 (13.7)		< 0.001
Routinely performs RCT^d^ on:							
Premolars	139	95%	95	92%	44	100%	0.1
Molars	116	79%	72	71%	44	100%	< 0.001
RCT characteristic	*N* = 1698		*N* = 778		*N* = 920		
RCT difficulty ratings (DR) reported by the treating practitioner							
Pre‐RCT DR: mean (SD)	3.92 (2.18)		3.90 (2.18)		3.93 (2.18)		0.9
Post‐RCT DR: mean (SD)	3.77 (2.29)		3.58 (2.34)		3.94 (2.24)		< 0.001
Post‐RCT minus Pre‐RCT DR: mean (SD)	‐0.14 (1.45)		−0.32 (1.59)		0.01 (1.30)		< 0.001
Pre‐RCT CDAF reasons reported by the treating practitioner							
Molar tooth	1019	60%	333	43%	686	75%	< 0.001
Calcifications present within pulp	417	25%	121	16%	296	32%	< 0.001
Longest > 21 mm cusp tip to apex	411	24%	204	26%	207	23%	0.14
Crown restoration present	400	24%	108	14%	292	32%	< 0.001
Limited ability open mouth	251	15%	125	16%	126	14%	0.03
Not all root canals clearly visible	228	13%	77	10%	151	16%	0.08
Curvature substantial or S‐shaped	190	11%	56	7%	134	15%	0.2
Gag reflex	67	4%	37	5%	30	3%	0.1
Root resorption	43	3%	17	2%	26	3%	0.2
Incomplete root development	6	< 1%	2	0%	4	0%	0.2
Other difficulty	171	10%	66	8%	105	11%	0.3
Number of reasons difficult: mean (SD)	1.89 (1.40)		1.47 (1.27)		2.24 (1.40)		< 0.001

^a^ *p*‐value: Fisher's exact for dentist categorical variables; Wilcoxon rank sum for continuous variables; differences in CDAF reasons are adjusted for clustering of patients within practitioner using generalised estimating equations (GEE) and whether tooth was a molar.

^b^ Other race‐ethnicity: 16 were Asian, 11 were African‐American, 10 were of other or multi‐race, and 3 were Hispanic.

^c^ Other practice settings: 14 were associates of a private practice, 13 were members of a preferred provider organisation, 9 in Academic, and 3 were in public or federal practices.

^d^ Missing for 6.

### 4.2 Patient Demographics

The mean (SD) age of patients was 48.3 (15.8) years and the median (IQR) was 49 (36–61) years. The majority (59%) were female, non‐Hispanic white (69%), had some form of dental insurance (76%), and 46% had a bachelor's degree or higher level of education. Endodontists treated marginally older patients, more women, and more educated patients (data previously published by Mungia et al. (2025)).

### 4.3 Tooth Types

Of the teeth treated, 12% were anterior, 27% premolars, and 60% molars (not shown in table). Endodontists treated a higher proportion of molars than GDs (75% vs. 43%, *p* < 0.001).

### 4.4 Difficulty Rating

The mean (SD) difficulty rating (DR) prior to RCT was 3.92 (2.18) and the median (IQR) was 4.0 (2.0–5.0), and after RCT the mean was 3.77 (2.29) and the median was 3.0 (2.0–5.0). Pre‐ and post‐RCT DR were highly correlated (*r* = 0.79, *p* < 0.001). There was no difference in the pre‐RCT DR between endodontists and GDs. Endodontists rated the post‐RCT case difficulty higher than GDs (3.94 vs. 3.58; *p* < 0.001), but their mean post‐RCT ratings were not different from their mean pre‐RCT ratings. There was a small but significant difference in pre‐RCT and post‐RCT DR (mean difference: −0.14 [SD = 1.45], *p* < 0.001). This was reported solely among GDs (*p* < 0.001); there was no difference among endodontists (Table 1).

### 4.5 Case Difficulty Assessment Form

CDAF (Table 1): in the Pre‐RCT assessment, the most frequently anticipated difficulties were the tooth being a molar (60%), calcifications within the pulp chamber (25%), when the longest cusp tip to apex distance was > 21 mm (24%), when a crown was present (24%), when there was limited mouth opening (15%), when canals were not all clearly visible (13%), and when there was a substantial curvature or the canal was S‐shaped (11%). Each of these items was more common among molars (*p* < 0.001, results data not shown). After adjusting for tooth type and clustering of patients within practitioner, pulp chamber calcifications and the presence of a crown were more commonly reported by endodontists (*p* < 0.001 for each). The mean (SD) number of pre‐RCT CDAF items was 1.89 (1.40), which was higher for endodontists than GDs (*p* < 0.001).

Among patients with a DR of 3 or higher, pre‐ and post‐RCT, the average agreement between pre‐ and post‐RCT assessment was 90% for the top 6 most frequently reported items. Among GDs, those who routinely performed RCTs on molars cited more items for RCT being difficult (*p* < 0.001, not shown).

### 4.6 Correlation Between Pre‐RCT DR and Number of Items Pre‐RCT From CDAF

There was a significant correlation between pre‐RCT DR and the pre‐RCT number of items reported in the CDAF (*r* = 0.57; *p* < 0.001). The correlation was higher among endodontists (*r* = 0.63, *p* < 0.001) than GDs (*r* = 0.52, *p* < 0.001).

### 4.7 Potential Impact of CDAF Items on DR

The potential impact of a specific CDAF item on the overall DR was determined by calculating the mean DR rating when the CDAF item was cited and compared to the mean DR rating when that CDAF item was not cited. The largest mean difference was found for canal visibility (not all canals were visible, mean difference 2.23), followed by calcifications within the pulp chamber (1.77), substantial curvature (1.76), gag reflex (1.64), limited ability to open the mouth (1.40), molar (1.28), and presence of a crown (1.10) (all *p* < 0.001). Only incomplete root development had no significant impact on DR. All the items cited above as having a significant impact on DR, except root resorption, retained a significant impact when assessed jointly, viz., all in one model (Table 2).

**TABLE 2:** Impact on pre‐RCT difficulty rating (DR) due to the specific reason cited on the pre‐RCT case difficulty assessment form (CDAF).

Pre‐RCT CDAF reasons cited	Specific pre‐RCT CDAF reason cited				Difference in mean pre‐RCT DR due to the specific pre‐RCT CDAF reason		
	Yes		No				
	Mean DR	SD	Mean DR	SD	Mean	SD	*p* ^a^ ^s^
Not all root canals clearly visible	5.86	1.81	3.63	2.08	2.23	2.04	< 0.001
Calcifications present within pulp	5.26	1.94	3.49	2.08	1.77	2.04	< 0.001
Curvature substantial or S‐shaped	5.48	1.96	3.73	2.13	1.76	2.11	< 0.001
Gag reflex	5.49	2.08	3.86	2.16	1.64	2.15	< 0.001
Limited ability to open mouth	5.09	2.00	3.72	2.15	1.40	2.12	< 0.001
Molar	4.43	2.02	3.15	2.17	1.28	2.08	< 0.001
Crown restoration present	4.79	2.01	3.66	2.16	1.10	2.13	< 0.001
Root resorption	4.64	2.18	3.91	2.18	0.73	2.17	0.02
Longest > 21 mm cusp tip to apex	4.43	2.21	3.77	2.15	0.68	2.16	< 0.001
Incomplete root development	3.33	1.97	3.92	2.18	−0.59	2.18	0.6

Abbreviation: SD, standard deviation.

^a^ *p*‐value from Wilcoxon rank sum test.

### 4.8 Complications During RCT

**FIGURE 2:**
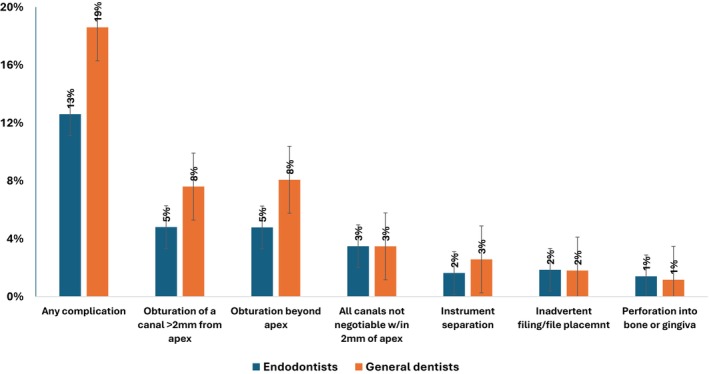
Frequency distribution of complications during nonsurgical root canal treatment: endodontists versus general dentsists.

Six complications were identified; four were reported upon completion of the RCT, and two were identified from the length of obturation reported separately from the other complications. Complications were identified in 16% (*n* = 273) of patients, of whom 75% (*n* = 205) had only one complication (not shown). The types of complications included the length of obturation material in at least one canal beyond the apex (*n* = 98; 6%) or > 2 mm from the apex (*n* = 101; 6%), one or more canals not negotiable within 2 mm of the apex (*n* = 59; 3%), separation of the instrument in canal space (*n* = 35; 2%), inadvertent filing/file placement past the root apex (*n* = 31; 2%), and perforation (from chamber or canal) into bone or gingival tissue (*n* = 22; 1%). Complications were less frequent among endodontists compared with GDs (13% vs. 19%, *p* < 0.001) (Figure 2).

### 4.9 Associations Between Pre‐RCT CDAF Items and Each Complication During the Subsequent RCT

All associations below have also been adjusted for whether the dentist was an endodontist. For the complication ‘length of obturation material in at least one canal > 2 mm short of the radiographic apex’, both ‘presence of a crown restoration’ (OR = 0.5, 95% CI: 0.3, 0.9; *p* = 0.01) and ‘Not all root canals clearly visible pre‐RCT’ (OR = 2.7, 95% CI: 1.6, 4.4; *p* = 0.007) were independently predictive of this complication (Table 3).

**TABLE 3:** Associations between pre‐RCT CDAF reasons stated and each complication during the subsequent RCT procedure.

Reason(s) the RCT was difficult, based on the pre‐RCT CDAF	Individual^a^		Adjusted for whether endodonist^b^		Adjusted^c^		
	Odds ratio	*p*	Odds ratio	*p*	Odds ratio	95% confidence interval	*p* ^d^
Outcome^e^: length of obturation material: a canal ≥ 2 mm from radiographic apex (*n* = 101)							
Molar	1.6	0.03	1.9	0.008	1.7	1.0, 2.7	0.046
Limited ability to open mouth	1.7	0.09	1.7	0.09	1.2	0.7, 2.3	0.5
Gag reflex^f^	2.6	0.04	2.6	0.046	2.0	0.9, 4.4	0.15
Crown restoration present	0.6	0.04	0.7	0.06	**0.5**	**0.3, 0.9**	**0.01**
Calcifications present within pulp chamber	1.5	0.04	1.8	0.02	1.5	1.0, 2.3	0.07
Longest root > 21 mm cusp tip to apex	0.8	0.3	0.8	0.3	Not entered		
Curvature substantial or S‐shaped	1.5	0.2	1.7	0.2	Not entered		
Not all root canals clearly visible	2.7	0.004	3.0	0.003	**2.7**	**1.6, 4.4**	**0.007**
Outcome: all canals not negotiable within 2 mm apex (*n* = 59)							
Molar	2.3	0.01	2.5	0.02	1.9 0.9, 4.2 0.08		
Limited ability to open mouth	1.1	0.8	1.1	0.9	Not entered		
Crown restoration present	1.3	0.3	1.3	0.3	Not entered		
Calcifications present within pulp chamber	3.5	0.003	3.7	0.003	**2.8**	**1.5, 5.4**	**0.01**
Longest root > 21 mm cusp tip to apex	0.8	0.4	0.8	0.4	Not entered		
Curvature substantial or S‐shaped	2.0	0.2	2.1	0.1	Not entered		
Not all root canals clearly visible	3.5	0.005	3.5	0.004	**2.5**	**1.4, 4.4**	**0.02**
Outcome: separation of instrument in canal space (*n* = 35)							
Molar	2.8	0.005	3.5	0.002	**2.8 1.2, 6.3 0.01**		
Limited ability to open mouth	1.0	0.9	0.9	0.9	Not entered		
Crown restoration present	0.8	0.7	0.9	0.8	Not entered		
Calcifications present within pulp chamber	2.1	0.08	2.4	0.05	2.0	0.9, 4.5	0.1
Longest root > 21 mm cusp tip to apex	1.6	0.2	1.6	0.2	Not entered		
Curvature substantial or S‐shaped	3.3	0.04	3.8	0.04	3.0	1.3, 6.9	0.06
Not all root canals clearly visible	1.1	0.8	1.2	0.8	Not entered		
Outcome: perforation into bone or gingival tissue (*n* = 22)							
Molar	3.3	0.05	3.4	0.09	2.8 0.7, 11.6 0.1		
Limited ability to open mouth	1.9	0.5	1.9	0.5	Not entered		
Crown restoration present	1.3	0.7	1.2	0.8	Not entered		
Calcifications present within pulp chamber	2.2	0.3	2.1	0.4	Not entered		
Longest root > 21 mm cusp tip to apex	0.2	0.008	0.2	**0.01**	**0.1**	**0.02, 0.8**	**0.005**
Curvature substantial or S‐shaped	3.9	0.08	3.8	0.08	4.1	1.5, 10.8	0.07
Not all root canals clearly visible	1.8	0.4	1.7	0.4	Not entered		

Abbreviation: CDAF, case difficulty assessment form.

^a^ Adjusted only for clustering of patients within practitioner with generalised estimating equations (GEE).

^b^ Adjusted for clustering of patients within practitioner with GEE and whether the dentist was an endodontist.

^c^ All variables with *p* < 0.1 when assessed individually, i.e., when only adjusted for clustering, are included. GEE used.

^d^ Type 3 GEE Analysis *p*‐value. *p* < 0.05 are bold.

^e^ Excludes 98 with canal beyond radiographic apex and 33 missing data on length of obturation.

^f^ Gag reflex was too rare a CDAF reason to assess with other complications, causing the models not to converge.

For the complication ‘all canals not negotiable within 2 mm of the apex’, the ‘presence of calcifications in the pulp chamber’ (OR = 2.8, 95% CI: 1.5, 5.4; *p* = 0.01) and ‘not all root canals being clearly visible’ (OR = 2.7, 95% CI: 1.6, 4.4; *p* = 0.007) were independently predictive.

For the complication ‘separation of instrument in canal space’, only molar (OR = 2.8, 95% CI: 1.5, 5.4; *p* = 0.01) was independently predictive.

For the complication ‘perforation into bone or gingival tissue’, ‘only longest cusp tip to root apex length > 21 mm’ (OR = 0.1, 95% CI: 0.02, 0.8; *p* = 0.005) was independently predictive.

No CDAF item was associated with inadvertent filing/file placement past root apex (results data not shown).

## 5 Discussion

This study offers salient insights into the factors influencing the perceived and actual difficulty of RCT among GDs and endodontists. The recruitment process ensured a broad representation of patients and providers, allowing for meaningful comparisons between general dentists and endodontists. Using a version of the CDAF that was modified by an AAE special committee so that it would fit the time requirements of practice‐based research, we explored how practitioners assessed endodontic case difficulty and how these assessments might change before and after RCT. Furthermore, a rating system (DR) was used to enhance the assessment of RCT difficulty. This approach parallels the scoring systems used in other surgical fields (Ban et al. 2014) where difficulty ratings guide practitioners in navigating varying levels of procedural complexity. By incorporating a DR system with the CDAF, our goal was to provide a more nuanced evaluation of RCT difficulty. This dual‐assessment approach also offers a way to validate the CDAF scores and examine how practitioners' subjective DRs align with objective case parameters, thereby enhancing the overall predictive accuracy and effectiveness of endodontic treatment planning. To our knowledge, this is the first study that has investigated relationships between case complexity and procedural errors within a practice‐based context.

The CDAF revealed that calcifications in the pulp chamber, long canals, full coverage coronal restorations, and limited visibility of canals were the most frequent challenges during RCT. Difficulties with canal visibility were associated with a higher likelihood of obturation material being > 2 mm short of the apex. Pulp chamber calcifications and poor canal visibility were significantly associated with a higher likelihood of canals being non‐negotiable, and longer canal lengths were inversely correlated with perforations. These findings provide insight into specific procedural risks and suggest that certain anatomical features may either exacerbate or mitigate particular complications. Pre‐RCT DRs correlated strongly with post‐RCT ratings, suggesting the accuracy of initial assessments. Endodontists reported more difficulty items and higher post‐RCT DRs than GDs, likely reflecting the exposure of endodontists to more‐complex cases. A stronger correlation was found between pre‐RCT DRs and the number of CDAF items among endodontists. Furthermore, the lack of a difference between pre‐ and post‐RCT DR compared to GDs suggests that endodontists' experiences may have provided them with a more‐nuanced pre‐RCT understanding of case complexity.

Our study's 3.5% incidence of non‐negotiable canals was much lower than the 8.9% reported by Yousuf et al. (2015). This difference is likely due in part to advances in canal negotiation techniques and improved instrumentation protocols since Yousuf's study. Our study reported that 5.9% of obturations were shorter by ≥ 2 mm and 5.8% of obturations extended beyond the apex, which was lower than the 8.9% of underfilled canals and 22.7% of overfilled canals previously reported (Yousuf et al. 2015). These differences might indicate improved obturation techniques over time and possibly reflect better materials, or enhanced training and protocols; particularly among endodontists (*p* = 0.02 for both). Furthermore, the incidence of instrument separation was 2.1%, aligning with the 2.3% reported by Gomes et al. (2021). Perforation into bone or gingiva occurred at a rate of 1.3% in our study, which is within the range of 0.6%–17.6% reported by Sarao et al. (2020). The wide variability in the literature underscores the challenges associated with preventing perforations and suggests that factors such as technique, experience, and case complexity could influence outcomes. The lower complication rate among endodontists may stem from their specialised training, which equips them to handle complex cases with greater precision. Additionally, their higher case volume and familiarity with challenging anatomical variations likely contribute to their ability to anticipate and mitigate complications more effectively than general dentists. Also, a PREDICT study comparing clinical approaches between GDs and endodontists revealed several differences between clinicians, such as higher odds of using magnification > 5×, rubber dam isolation, electronic apex locators, and complex obturation techniques by endodontists (Nosrat et al. 2025). These findings can also be related to lower rates of procedural complications among endodontists compared to GDs.

As the post‐RCT CDAF was only completed if the post‐RCT DR was 3 or higher, this limited some comparisons of CDAF items. The procedural complications were rare (as they should be), which limited the ability to assess whether specific items were associated. There was no power to assess interactions of CDAF items and complications (e.g., molar and substantial curvature or S‐shaped with separation of instrument in canal space).

This study has several strengths, including a large sample size, national representation, varied practice settings, and practitioner and patient demographics. While we cannot assert that Network dentists are entirely representative of dentists at large (due to unmeasured characteristics, such as desire to participate in clinical research), we can state that they have much in common with dentists at large, while also offering substantial diversity in these characteristics. This assertion is warranted because: (1) substantial percentages of Network dentists are represented in the various response categories of the characteristics in the Enrollment Questionnaire; (2) findings from several Network studies document that Network dentists report patterns of diagnosis and treatment that are similar to patterns determined from non‐network GDs; and (3) the similarity of Network dentists to non‐Network dentists using comparisons to national survey data (Cunha‐Cruz et al. 2023; Makhija, Gilbert, Rindal, et al. 2009, 2009; Norton et al. 2014; Gilbert, Gordan, et al. 2015; Gilbert, Riley, et al. 2015). However, differences in expertise, case volume, access to specialised equipment, and procedural approaches (Nosrat et al. 2025) between general dentists and endodontists could impact treatment outcomes, making direct comparisons challenging. Additionally, payment models may play a role in treatment approaches and complication rates, as financial incentives and reimbursement structures could influence clinical decisions. A more detailed analysis of how these factors intersect would provide further insight into their impact on study findings. Future research could explore patient perspectives on treatment difficulty and post‐procedural outcomes, as well as assess how findings could inform educational curricula for dental practitioners, ensuring improvements in training and patient care.

## 6 Conclusions

This study highlights the importance of pre‐RCT assessments and the identification of technical challenges such as calcifications in the pulp chamber, long canals, the presence of full coverage coronal restorations, and limited visibility of canals in RCT; and the significant role that practitioner experience and expertise play in successful endodontic treatment.

## Author Contributions

A.S.L., R.M., E.F., D.R.N., G.H.G.: contributed to conception and design; acquisition, analysis, or interpretation of data; drafted the manuscript; critically revised the manuscript for important intellectual content; gave final approval; agree to be accountable for all aspects of the work in ensuring that questions relating to the accuracy or integrity of any part of the work are appropriately investigated and resolved. E.W.N.L.: contributed to conception and design; interpretation of data; critically revised the manuscript for important intellectual content; gave final approval; agreed to be accountable for all aspects of the work in ensuring that questions relating to the accuracy or integrity of any part of the work are appropriately investigated and resolved. S.N., R.S.R., A.N.: contributed to interpretation of data; critically revised the manuscript for important intellectual content; gave final approval; agreed to be accountable for all aspects of the work in ensuring that questions relating to the accuracy or integrity of any part of the work are appropriately investigated and resolved.

## Conflicts of Interest

The authors declare no conflicts of interest.

## Data Availability

The data that support the findings of this study are available from the corresponding author upon reasonable request.
